# AI-assisted peripheral immune profiling reveals unconventional lymphocyte signatures associated with prognosis in soft tissue sarcoma patients

**DOI:** 10.3389/fimmu.2025.1677408

**Published:** 2025-10-27

**Authors:** Jani Sofia Almeida, Luana Madalena Sousa, Patrícia Couceiro, Vera Alves, Joana Rodrigues, Ruben Fonseca, Paulo Freitas-Tavares, Manuel Santos-Rosa, José Manuel Casanova, Paulo Rodrigues-Santos

**Affiliations:** ^1^ Laboratory of Immunology and Oncology, Center for Neurosciences and Cell Biology (CNC), University of Coimbra, Coimbra, Portugal; ^2^ Center for Investigation in Environment, Genetics and Oncobiology (CIMAGO), University of Coimbra, Coimbra, Portugal; ^3^ Coimbra Institute for Clinical and Biomedical Research (iCBR), University of Coimbra, Coimbra, Portugal; ^4^ Center for Innovation in Biomedicine and Biotechnology (CIBB), University of Coimbra, Coimbra, Portugal; ^5^ Clinical and Academic Centre of Coimbra (CACC), Coimbra, Portugal; ^6^ Institute of Immunology, Faculty of Medicine (FMUC), University of Coimbra, Coimbra, Portugal; ^7^ Tumor Unit of the Locomotor Apparatus (UTAL), University Clinic of Orthopedics, Orthopedics Oncology Service, Coimbra Local Health Unit (ULSC), Coimbra, Portugal

**Keywords:** soft tissue sarcoma, unconventional lymphocytes, γδ T cells, NKT-like cells, unsupervised clustering, multiparametric flow cytometry

## Abstract

Immunotherapy has reshaped the treatment of several cancers, yet patient responses remain highly variable, partly due to differences in immune competence. In soft tissue sarcomas (STS), the immune landscape is poorly characterized, limiting the development of prognostic markers and immune-based therapeutic strategies. This study aimed to comprehensively profile circulating and tumor-infiltrating cytotoxic lymphocyte populations in STS. Peripheral blood from patients and healthy donors was analyzed by multiparametric flow cytometry combined with AI-assisted unsupervised clustering, enabling the identification of both conventional and unconventional subsets. In a pilot cohort, tumor-infiltrating lymphocytes were evaluated using the same approach to explore systemic–local immune compartmentalization. STS patients displayed systemic immune imbalance with increased CD8^+^ T cells and reduced NK cells and CD161^+^ CD8^+^ T cells, consistent with overall immunosuppression. Several unconventional populations showed prognostic associations: elevated CD8^+^ γδ T cells and CD4^+^ NKT-like cells correlated with poorer survival, whereas CD8^+^ NKT-like cells were enriched in immune-competent patients and linked to better outcomes, suggesting potential protective functions. Pilot tumor analyses identified γδ NKT-like cells that were nearly absent from circulation, suggesting their selective enrichment within the tumor microenvironment. Together, these findings highlight the contribution of rarely profiled cytotoxic lymphocytes to systemic immune fitness and disease outcome in STS. Importantly, despite clinical and histological heterogeneity, patients showed consistent immune alterations, suggesting shared immunological features across STS subtypes. While limited by small tumor sample size and lack of functional assays, this study provides proof-of-concept that immune-based profiling can uncover novel prognostic markers and candidate populations of therapeutic relevance. Future work in larger, longitudinal cohorts, coupled with functional characterization, will be essential to validate these subsets and to define their role in STS immune surveillance and responsiveness to immunotherapy.

## 1 Introduction

The introduction of immunotherapy has brought new hope and a powerful alternative for treating cancer (1, 2). However, even among patients with the same tumor type, particularly those expected to respond, clinical outcomes remain highly variable, and many patients fail to benefit (3). This variability has fueled growing interest in the concept of immune fitness or competence as a potential predictor of treatment response (4, 5). Yet, despite its conceptual appeal, how to assess immune function in a practical, accessible, and clinically meaningful way remains an open challenge.

In this context, peripheral blood (PB) has emerged as a promising, minimally invasive alternative to tumor biopsies for capturing systemic immune states and enabling longitudinal immune monitoring (6, 7). While the tumor microenvironment (TME) provides valuable insights into local immune dynamics, it is not always feasible to access. Consequently, efforts to identify clinically informative peripheral immune signatures, capable of reflecting tumor burden, metastatic potential, or response to therapy, have intensified, driven by the ease and repeatability of blood sampling (8–10). Establishing such signatures is now a central objective in the advancement of immune profiling in oncology.

One of the key advantages of immune-based stratification is its potential to transcend the biological and histological heterogeneity that characterizes cancer (11). Immune signatures may capture clinically relevant features that are not necessarily reflected in tumor classification, offering a functional layer of patient characterization that could guide treatment decisions. This raises the possibility that patients, despite underlying molecular or histological differences, could be stratified and treated according to their immune capacity to mount an anti-tumor response.

Our group previously explored this concept in a cohort of soft tissue sarcoma (STS) patients, a particularly highly heterogeneous group of malignancies (12). Through a comprehensive immunoprofiling approach that included nearly 300 parameters (circulating immune cells, immune-related gene expression, and soluble plasma analytes) we identified three distinct peripheral immune profiles that correlated with clinical outcomes (13). Patients classified as “immune high”, characterized by elevated levels of activation/cytotoxic markers (e.g., CD69, CD40L, GZMB, NCR2) and low levels of immunosuppressive/inflammatory variables (e.g., PMN-MDSC, ARG1, GR, and IL17-A), showed improved survival. In contrast, “immune low” patients displayed a suppressive, inflammatory and poorly cytotoxic profile, and were associated with poorer outcome, while an intermediate group showed a mixed immune phenotype and moderate survival.

Although these findings underscored the prognostic relevance of immune profiling, it is recognized that such broad, multi-parametric analyses are not yet feasible in clinical practice due to their complexity, cost, and response time. As a result, attention has shifted toward simpler circulating immune markers, such as the neutrophil-to-lymphocyte ratio (NLR) and monocyte-to-lymphocyte ratio (MLR), which have shown prognostic value across various cancers (14–16). However, while accessible, these metrics mainly reflect systemic inflammation and fail to capture the functional diversity and cytotoxic potential of immune cells critical for anti-tumor responses, such as NK cells, CD8^+^ T cells, and unconventional lymphocyte subsets.

To address the gap between comprehensive immune profiling and clinical feasibility, we used a well-characterized cohort of STS patients to identify cytotoxic immune cell populations with potential as accessible and informative biomarkers. Building on previously defined peripheral immune profiles (“immune high,” “intermediate,” and “immune low”), this study aimed to characterize circulating lymphocyte subsets that reflect systemic cytotoxic immune competence. Although the primary focus was on PB samples, we also examined tumor samples from a subset of STS patients to investigate immune differences between circulation and the TME. To maximize sensitivity and avoid overlooking rare but clinically relevant populations, we combined multiparametric flow cytometry with unsupervised, AI-assisted clustering (17–19). This strategy enabled the identification of both conventional and unconventional cytotoxic lymphocytes with prospective clinical relevance. Linking peripheral immune features to systemic immune competence and clinical outcomes advances the understanding of the immune landscape in STS and supports the identification of candidate biomarkers with translational potential.

## 2 Material and methods

### 2.1 STS patients and healthy donors

Between November 2020 and February 2023, PB samples and clinical data were collected at the Tumor Unit of the Locomotor Apparatus, University Clinic of Orthopedics, Orthopedic Oncology Service, Coimbra Local Health Unit, a designated European Reference Center for Adult STS Treatment. Eligible participants included adults (≥18 years) with a confirmed diagnosis of STS, excluding gastrointestinal stromal tumors. Patients with active viral or bacterial infections were excluded. A total of 29 PB samples from STS patients, classified into three immune subgroups: P1 (“immune high”, n=9), P2 (“immune intermediate”, n=8), and P3 (“immune low”, n=12), 25 PB samples from healthy donors as control (Ctrl) group and 9 STS tumor tissue samples, were included in the study. The subgroup classification was based on previously published STS immune profiles: P1 (“immune high”) with predominant cytotoxic markers and better survival; P3 (“immune low”) with suppressor markers and worse survival; and P2 showing mixed profiles with intermediate outcomes (13). All procedures involving human participants followed the ethical standards outlined in the Declaration of Helsinki. Written informed consent was obtained from all individuals after a detailed explanation of the study’s purpose and procedures. Ethical approval was obtained from the Ethics Committees of the Coimbra Hospital and University Centre (CHUC-021-19) and the Faculty of Medicine, University of Coimbra (CE-018/2021). Demographic and clinical data are summarized in **Supplementary Table S1**.

### 2.2 Preparation of single-cell suspensions from tumor tissue

Tumor tissue samples (1–2 cm²) were collected in 1x Dulbecco′s Phosphate Buffered Saline (D-PBS, Sigma-Aldrich, St. Louis, MO, USA) immediately after surgical excision. To eliminate PB contamination, tissues were first rinsed thoroughly with 1x D-PBS. Subsequently, samples were finely sliced into ~1 mm² fragments using a sterile petri dish. These fragments were then transferred into 5 mL Eppendorf tubes containing 2.5 mL of a 1x Collagenase/Hyaluronidase solution (STEMCELL Technologies, Vancouver, BC, Canada) prepared in Dulbecco′s Modified Eagle′s Medium (DMEM, Sigma-Aldrich, St. Louis, MO, USA) and incubated at 37 °C with agitation for 1 hour to overnight, depending on the tissue origin. Following enzymatic digestion, the dissociated cell suspensions were transferred into 50 mL conical tubes containing DMEM supplemented with 10% (v/v) of 7.5% Bovine Serum Albumin (BSA) Fraction V (Gibco™, Thermo Fisher Scientific, Waltham, MA, USA). Cells were then centrifuged at 450 x g for 5 minutes. The supernatant was discarded, and the pellet resuspended in 1 mL of DMEM with 10% BSA for white blood cell quantification using a DXH 500 hematology analyzer (Beckman Coulter, Pasadena, CA, USA).

### 2.3 Multiparametric flow cytometry sample preparation and acquisition

Fresh PB samples collected in EDTA-treated tubes were processed for immunophenotyping by multiparametric flow cytometry. Initial whole blood counts were performed using the DxH 500 hematology analyzer (Beckman Coulter, Pasadena, CA, USA). For extracellular staining, 100 µL of whole blood or up to 1 x 10^6^ white blood cells were incubated with fluorochrome-conjugated monoclonal antibodies for 15 minutes at room temperature in the dark. Red blood cell lysis was then carried out using 2 mL of BD Lysing Solution (BD Biosciences, San Jose, CA, USA), with a 10-minute incubation at room temperature in the dark. Samples were centrifuged at 450 x g for 5 minutes, the supernatant discarded, and cells washed with 1x D-PBS. After a second centrifugation under the same conditions, the supernatant was removed, and cells were resuspended in 300 µL of 1x D- PBS for flow cytometry acquisition. Data were acquired on a BD FACSCanto II cytometer and analyzed using BD FACSDiva software (BD Biosciences, San Jose, CA, USA). Fresh tumor cell suspensions were stained using the same protocol, starting with 1 x 10^6^ white blood cells. A complete list of the antibodies used for both PB, and tumor samples is provided in **Supplementary Table S2**.

### 2.4 Unsupervised AI-assisted multiparametric flow cytometry data analysis

#### 2.4.1 Quality control and sample preprocessing

Flow cytometry files were imported into FlowJo^®^ software (v10.9, BD Biosciences, San Jose, CA, USA) for analysis. Quality control was performed using the FlowAI plugin (20), which filtered out abnormal events based on flow rate irregularities, signal stability, and fluorescence intensity range. Samples were annotated with metadata keywords to indicate group assignments, such as Ctrl, STS, STS immune profiles (P1, P2, P3), and STS tumor samples. For each individual sample, cells positive for CD3 and/or CD56 were identified following the gating strategy illustrated in **Supplementary Figure S3**. This gated population was then concatenated by group and the number of events reduced using the Downsample plugin for FlowJo^®^ software to equalize event counts across groups, minimizing bias due to varying sample sizes per group. For more details, see the official Downsample plugin page (https://www.flowjo.com/exchange/plugin/downsample). For multiparametric AI-assisted analysis, two separate concatenated files were created from the downsampled group files to maintain balanced event representation. One concatenated file combined PB samples from Ctrl and STS patients (100,000 total events, 50–000 events per Ctrl and STS, and the same event number per STS subgroups). The other concatenated file merged PB and tumor samples from STS patients (100,000 events, 50–000 events per PB and Tumor, and the same event number per STS groups). Each concatenated dataset was analyzed independently to investigate cellular phenotypes across sample groups.

#### 2.4.2 Dimensionality reduction and unsupervised clustering

Unsupervised clustering was conducted using the FlowSOM plugin (21), based on the expression of key cytotoxic lymphocyte lineage markers: CD3, CD4, CD8, CD56, CD161, and TCR γ/δ. FlowSOM was configured to generate eight metaclusters by grouping cells into self-organizing maps according to their expression profiles. To visualize cell distribution and interrelationships, t-SNE was applied using the built-in FlowJo^®^ plugin, with the following parameters: perplexity = 30, iterations = 1000, learning rate = 7000.

#### 2.4.3 Cluster annotation and visualization

Cluster Explorer plugin included in FlowJo^®^ software (v10.9, BD Life Sciences) was used to overlay FlowSOM-derived clusters onto the t-SNE map, enabling comprehensive visual assessment and manual annotation based on marker expression intensities. Heatmaps, histograms, and expression overlays were used to annotate the phenotypes of immune populations represented by each cluster. Group identification based on keywords is detailed in **Supplementary Figure S4** and was subsequently used to compare immune profiles between STS and Ctrl samples, among the STS immune profiles, P1 (“immune high”), P2 (“immune intermediate”), and P3 (“immune low”), and between PB and tumor samples from STS patients.

#### 2.4.4 Per-sample cluster frequency calculation

Cluster Explorer does not allow direct export of cluster frequencies per individual sample. Therefore, each of the eight annotated clusters was overlaid onto individual sample gates, defined by sample IDs via original metadata keywords. The frequencies of each cluster within individual samples were then exported from FlowJo^®^ for subsequent statistical analysis. This approach enabled precise determination of cluster distribution on a per-sample basis. For concatenated analyses, an equal number of events was randomly selected from CTRL and STS groups. Within STS subtypes, event numbers were further equalized across subtypes. Because patient numbers varied between subtypes, events were normalized at the group level to preserve comparability. Consequently, not all patients contributed equally to the concatenated dataset, and results from unsupervised concatenated analyses should be interpreted with caution when compared to per-sample analyses.

### 2.5 Statistical analysis

Exported cluster frequencies were used to compare immune profiles between Ctrl and STS PB samples, among STS immune profiles (P1, P2, P3), and between PB and tumor samples from STS patients. Statistical analysis and graph preparation were performed using GraphPad Prism v9.0.2 (GraphPad Software, San Diego, CA, USA). Mann–Whitney U tests were used for two-group comparisons with Holm-Šídák method to correct for multiple comparisons (PB from Ctrl vs PB from STS; PB from STS vs tumor from STS), and Kruskal–Wallis tests with Dunn’s *post hoc* correction were applied for multi-group comparisons. Adjusted *p*-values were considered. Survival analyses based on time-to-event data were carried out using IBM SPSS Statistics version 26.0 for Mac OS (IBM Corp, Armonk, NY, USA). Kaplan-Meier survival curves and log-rank tests were used to assess the impact of the studied parameters on patient survival. Time was defined as the duration from the sample collection date to either death or the end of the study (time after collection, TAC). Statistical significance was set at *p* < 0.05.

## 3 Results

To characterize the systemic cytotoxic immune landscape in STS and identify peripheral lymphocyte subsets with potential prognostic value, we analyzed flow cytometry data from 29 PB samples of STS patients and 25 healthy donors (control group, Ctrl). Building on previously defined immune profiles, “immune high” (P1, n = 9), “intermediate” (P2, n = 8), and “immune low” (P3, n = 12), patients were stratified accordingly to investigate associations between immune cell populations, systemic immune states, and clinical outcomes. To maximize resolution, AI-assisted unsupervised clustering and dimensionality reduction techniques were applied to identify both conventional and unconventional CD3^+^ and/or CD56^+^ lymphocyte populations. As a complementary analysis, tumor samples from a subset of STS patients (n = 9) were included to provide preliminary insight into the relationship between circulating and tumor-infiltrating immune populations.

### 3.1 STS exhibit distinct patterns of circulating CD3^+^ and/or CD56^+^ populations

**Figure 1:**
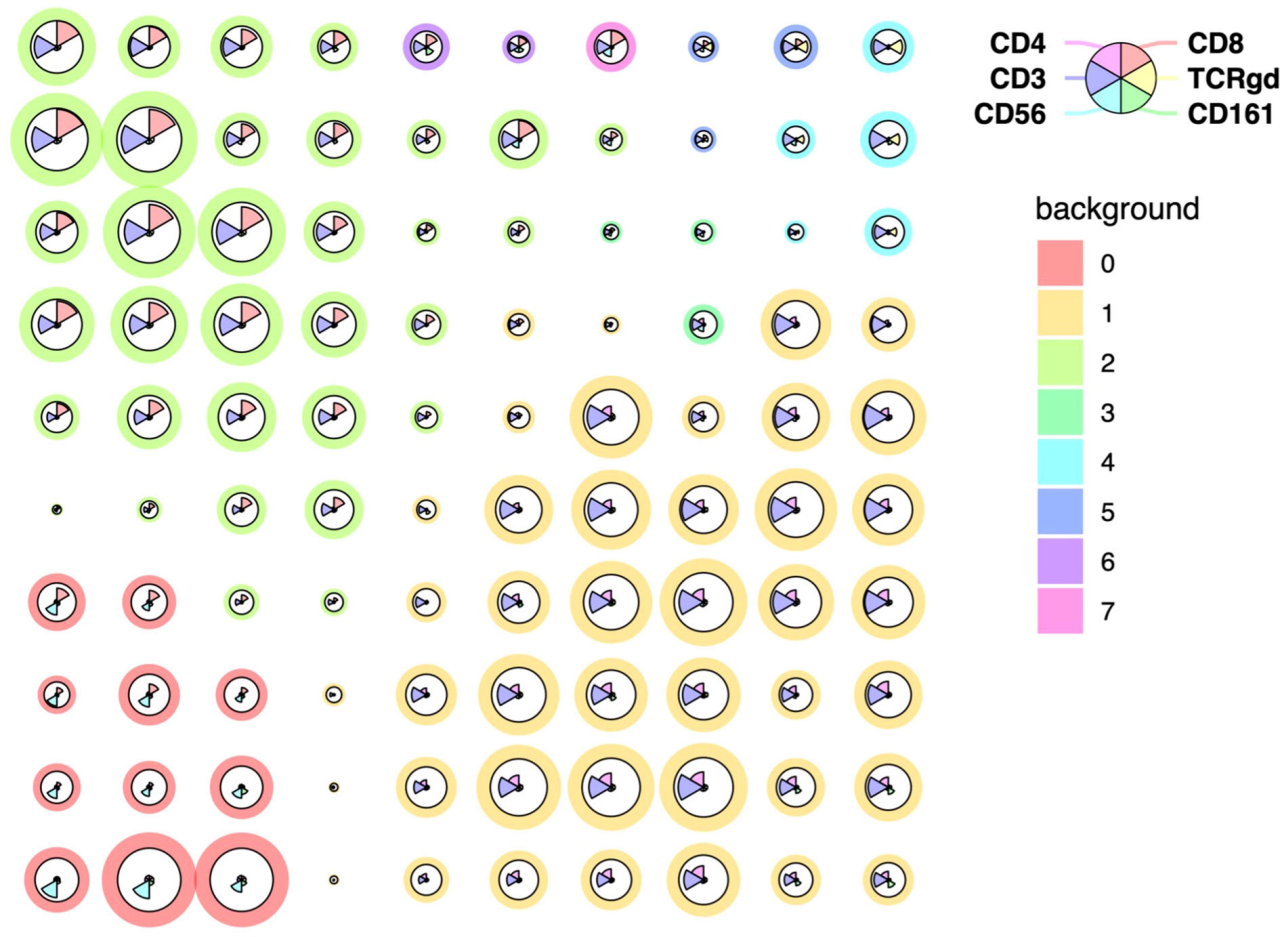
Unsupervised clustering and dimensionality reduction of PB CD3+ and/or CD56+ immune cell populations from STS and Ctrl samples. FlowSOM clustering of concatenated samples including only CD3^+^ and/or CD56^+^ lymphocytes. Cells were clustered based on the expression of CD3, CD4, CD8, CD56, CD161 and TCR γ/δ and grouped into eight metaclusters reflecting major immune populations indicated by background color scale (0 to 7). Each node represents a cluster, with size indicating relative abundance and color scale reflecting marker expression intensity.

Prior to unsupervised clustering, quality control of flow cytometry files was performed using the FlowAI plugin (20), which filtered out anomalous events. Additionally, samples were annotated with metadata keywords to indicate group assignments, such as Ctrl, STS, and STS immune profiles (P1, P2, P3). Next, all samples underwent manual analysis to identify the relevant cell populations, those expressing CD3 and/or CD56, selective for NK and T populations key mediators of cytotoxic anti-tumor responses (**Supplementary Figure S3**). CD3^+^ and/or CD56^+^ lymphocytes were concatenated into a single flow cytometry file, and cluster analysis was performed using the FlowSOM algorithm (21). This analysis generated eight metaclusters based on the expression of CD3, CD4, CD8, CD56, CD161, and TCR γ/δ (**Figure 1**).

**Figure 2:**
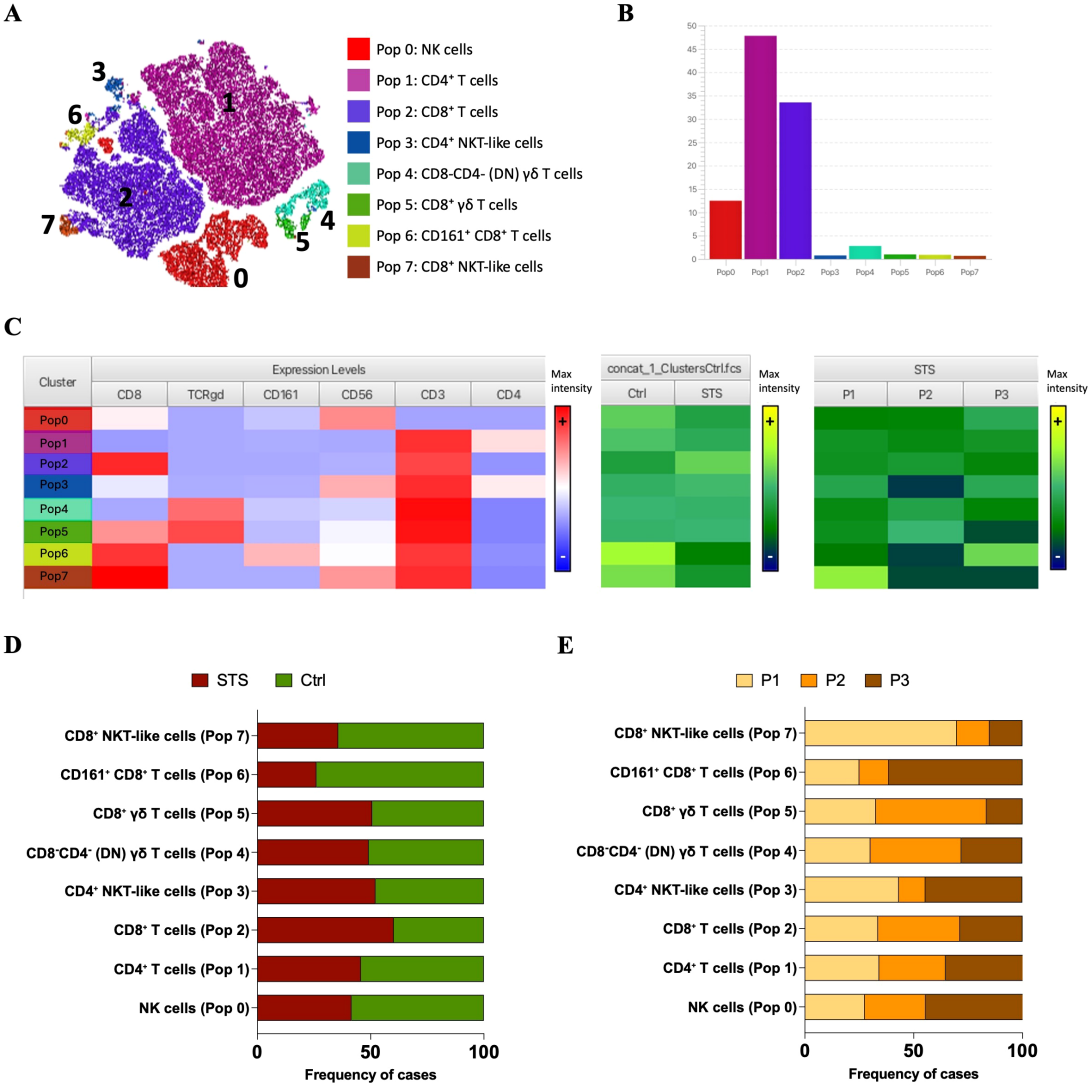
Cluster Explorer analysis reveals distinct immune cell populations in PB from STS and Ctrl samples. **(A)** t-SNE map displaying FlowSOM-defined clusters based on multiparametric marker expression. Each dot represents a single cell, with colors corresponding to FlowSOM-assigned clusters. **(B)** Bar plot showing the relative frequency of each identified immune population across all samples. **(C)** Cluster Explorer heatmap summarizing marker expression profiles per cluster. Rows represent individual clusters; columns represent surface markers. Color intensities represent median expression levels; (+) indicates higher intensity, whereas (–) indicates lower intensity. **(D)** Bar plot showing the distribution of STS and Ctrl samples across each immune cluster. **(E)** Bar plot showing the distribution of STS peripheral immunotypes (P1, P2, and P3) per cluster. *Legend: PB – peripheral blood; STS – soft tissue sarcoma; Ctrl – control; P1 – “immune high”; P2 – “immune intermediate”; P3 – “immune low”; Pop 0 – NK cells; Pop 1 – CD4^+^ T cells; Pop 2 – CD8^+^ T cells; Pop 3 – CD4^+^ NKT-like cells; Pop 4 – CD8-CD4- (DN) γδ T cells; Pop 5 – CD8^+^ γδ T cells; Pop 6 – CD161^+^ CD8^+^ T cells; Pop 7 – CD8^+^ NKT-like cells*.

To further explore the structure of FlowSOM-derived clusters, Cluster Explorer was employed for dimensionality reduction and annotation. The t-SNE map displayed the distribution of cells across the eight clusters identified in PB samples from STS and Ctrl groups (**Figure 2A**). Each cluster formed a distinct island in the 2D space, suggesting robust separation based on marker expression profiles. The corresponding bar plot summarizes the proportion of cells assigned to each cluster across the concatenated dataset, each cluster was defined as Pop 0 to Pop 7 (**Figure 2B**). Pop 1 and Pop 2 represented the most abundant populations, followed by Pop 0. Pop 3, 4, 5, 6 and 7 accounted for only a minor fraction of the total events.

To characterize the immune phenotypes represented by each cluster, the expression patterns of key surface markers was analyzed using the Cluster Explorer heatmap (**Figure 2C**). Each cluster exhibited a distinct marker expression profile, enabling the inference of putative immune cell identities (**Figures 2A–C**). Pop 0 displayed high CD56 expression and lacked CD3, suggesting a population of NK cells. Pop 1 and 2 expressed CD3 along with high levels of CD4 and CD8, respectively, consistent with conventional CD4^+^ and CD8^+^ T cells. Pop 3 co-expressed CD56, CD3, and CD4, likely corresponding to CD4^+^ NKT-like cells. Pop 4 showed high expression of CD3 and TCR γ/δ, with no expression of CD4 or CD8, suggesting a population of double-negative (DN) γδ T cells. In contrast, Pop 5 shared the same profile as Pop 4 but expressed CD8, indicating CD8^+^ γδ T cells. Pop 6 was distinguished from Pop 2 by the additional expression of CD161, suggesting CD161^+^ CD8^+^ T cells. Finally, Pop 7 differed from Pop 3 by expressing CD8 and lacking CD4, consistent with a population of CD8^+^ NKT-like cells.

To characterize the distribution of immune populations, it was analyzed the relative composition of each FlowSOM-defined cluster (Pop 0–Pop 7) based on the contribution of group samples. This cluster-centered perspective reflects the proportion of events from each group that make up 100% of each population. Stratification of STS and Ctrl group revealed distinct population distributions (**Figures 2C, D**). The most prominent differences were the higher contribution of STS-derived events to CD8^+^ T cells (Pop 5) and the lower contribution to CD161^+^ CD8^+^ T cells (Pop 6) and CD8^+^ NKT-like cells (Pop 7). In contrast, NK cells (Pop 0) and CD4^+^ T cells (Pop 1) showed a slight predominance of CTRL-derived events. Further stratification of the STS group according to immune profiles (P1–P3) revealed distinct distribution patterns (**Figures 2C, E**). P1-derived events were mainly associated with CD8^+^ NKT-like cells (Pop 7), whereas P2-derived events contributed predominantly to CD8^+^ γδ T cells (Pop 5) and DN γδ T cells (Pop 4). Conversely, P3-derived events were enriched in CD161^+^ CD8^+^ T cells (Pop 6) and NK cells (Pop 0). CD8^+^ T cells (Pop 5) had comparable contributions from P1 and P2, while CD4^+^ NKT-like cells (Pop 3) and CD4^+^ T cells (Pop 1) were similarly represented by P1- and P3-derived events.

### 3.2 STS show alterations in circulating lymphocytes: lower NK and CD161^+^ CD8^+^ T cells, and higher CD8^+^ T cells

**Figure 3:**
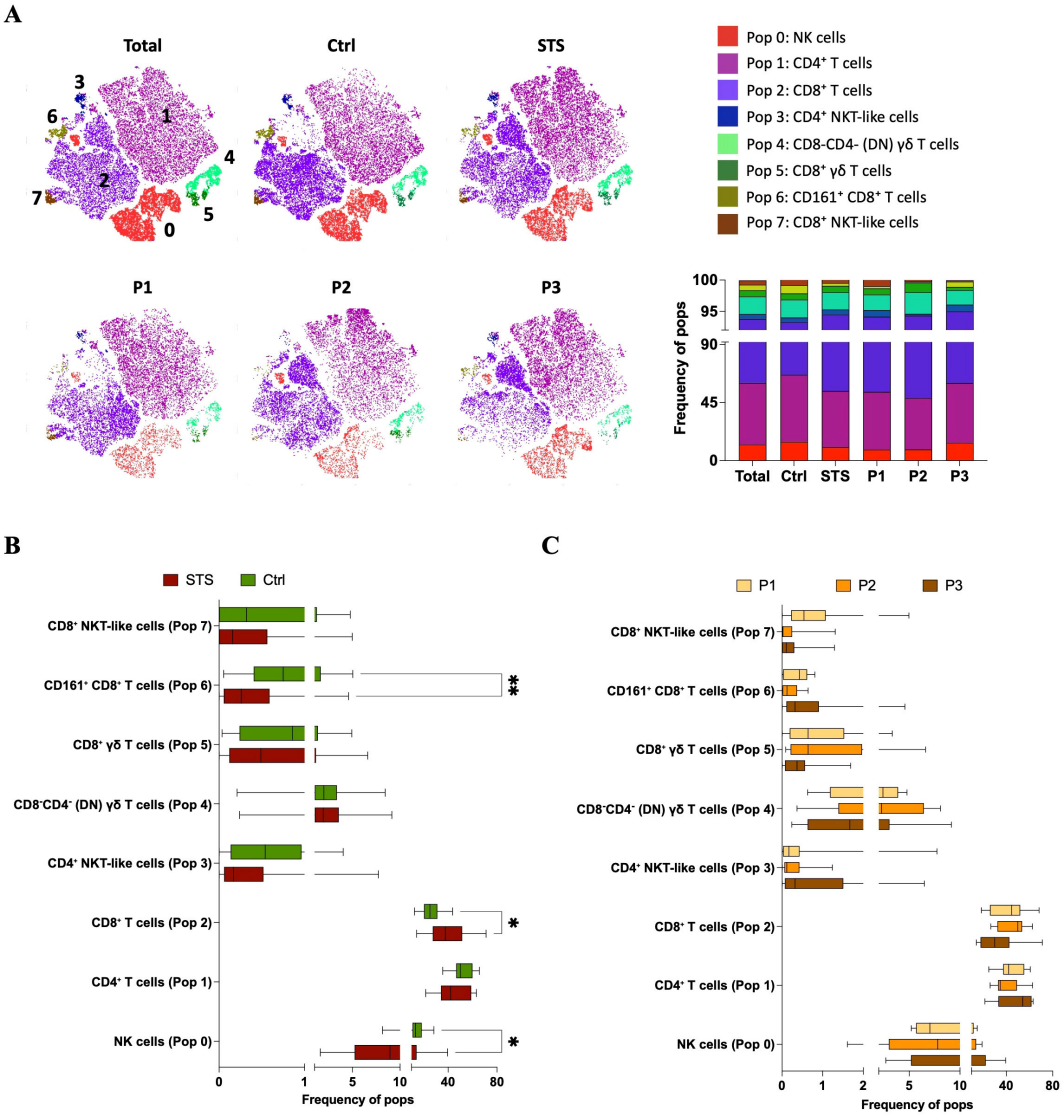
Sample-level comparison of immune cluster frequencies in PB reveals immunotype-specific alterations in STS. **(A)** t-SNE plots colored by FlowSOM-assigned clusters, representing: all samples combined (total), Ctrl samples, STS samples, and the three STS immunotypes (P1, P2, P3). Bar plot representation of FlowSOM-defined immune cluster frequencies in PB across analyzed groups. **(B)** Comparative analysis of cluster frequencies between STS and Ctrl samples. Statistical analysis was performed using the Mann–Whitney U test with Holm-Šídák method to correct for multiple comparisons. **(C)** Cluster frequency comparisons across STS immunotypes (P1, P2, and P3), using Kruskal–Wallis test with *post hoc* Dunn’s correction for multiple comparisons. Adjusted *p*-values are reported, with significance set at *p* < 0.05. Legend: PB – peripheral blood; STS – soft tissue sarcoma; Ctrl – control; P1 – “immune high”; P2 – “immune intermediate”; P3 – “immune low”; *p < 0.05; **p < 0.01.

Since Cluster Explorer does not support the direct export of cluster frequencies at the individual sample level, we performed a back-gating of the identified populations by sample using FlowJo^®^. This allowed for sample-level quantification within both the STS and Ctrl groups, including stratification by peripheral immune profiles (P1, P2, and P3) (13). **Figure 3A** shows a t-SNE map illustrating the distribution of the eight identified populations across study groups, alongside a bar graph displaying their relative frequency contributions within each group. Interestingly, in **Figure 3A** (bottom), total CD8^+^ and CD4^+^ T cells exhibited distinct distribution patterns across patient immune profiles, likely reflecting differences in underlying classical T cell subsets. Quantitative comparison at the individual sample level revealed that NK cells (Pop 0) and CD161^+^ CD8^+^ T cells (Pop 6) were significantly reduced in STS patients (Pop 0 = 8.96, 1.61 – 39.06; Pop 6 = 0.26, 0.00 – 4.58) comparing with Ctrl samples (Pop 0 = 13.30, 8.15 – 28.30, adj *p* = 0,017282; Pop 6 = 0.75, 0.05 – 5.04, adj *p* = 0.006274), whereas CD8^+^ T cells (Pop 2) was significantly increased in the STS group (Pop 2 = 37.80, 14.20 – 71.10) compared with Ctrl (Pop 2 = 25.20, 12.40 – 43.60, adj *p* = 0.026830) (**Figure 3B**). Further comparison among STS subgroups did not reveal significant differences, yet a trend was observed for higher levels of CD8^+^ NKT-like cells (Pop 7) in P1 patients (Pop 7 = 0.54, 0.00 – 4.98) comparatively with P2 (Pop 7 = 0.02, 0.00 – 0.31, adj *p* = 0.0727) and P3 (Pop 7 = 0.10, 0.00 – 1.29, adj *p* = 0.1444) patients (**Figure 3C**). It is important to note that, for CD161^+^ CD8^+^ T cells, the association with the P3 immunotype observed in the cluster-based analysis was not evident at the individual sample level. In fact, P1 patients displayed a higher median frequency than P3, although variability was greater in P3, as reflected by a higher standard deviation. Together with the unequal number of patients per group, this explains the discrepancy between the two analytical approaches. For the other populations, distribution patterns were consistent across both analyses.

### 3.3 Circulating γδ T and NKT-like subpopulations show prognostic relevance in STS

Despite the lack of statistically significant differences in immune cell cluster frequencies across STS immune profiles previously linked to survival, it was hypothesize that specific lymphocyte subsets could still possess prognostic relevance at the individual patient level. Since the patients included in this analysis represent a subset of those from a previously published study (13), a survival analysis across the defined immune profiles (P1, P2, P3) was performed within this subgroup to assess whether the association with clinical outcomes remained consistent. The impact of peripheral immunotypes on clinical outcomes was assessed using time-to-event from the date of sample collection to either the date of death or the end of follow-up, referred to as time after collection (TAC). Although no statistically significant differences were observed, likely due to the small sample size, the survival trend previously reported was maintained: P1 patients showed better survival, P3 patients had the poorest outcomes, and P2 patients displayed intermediate survival (**Supplementary Figure S5**). The difference between P1 and P3 patients approached statistical significance (*p* = 0.074), further supporting the relevance of these immune profiles to clinical prognosis.

**Figure 4:**
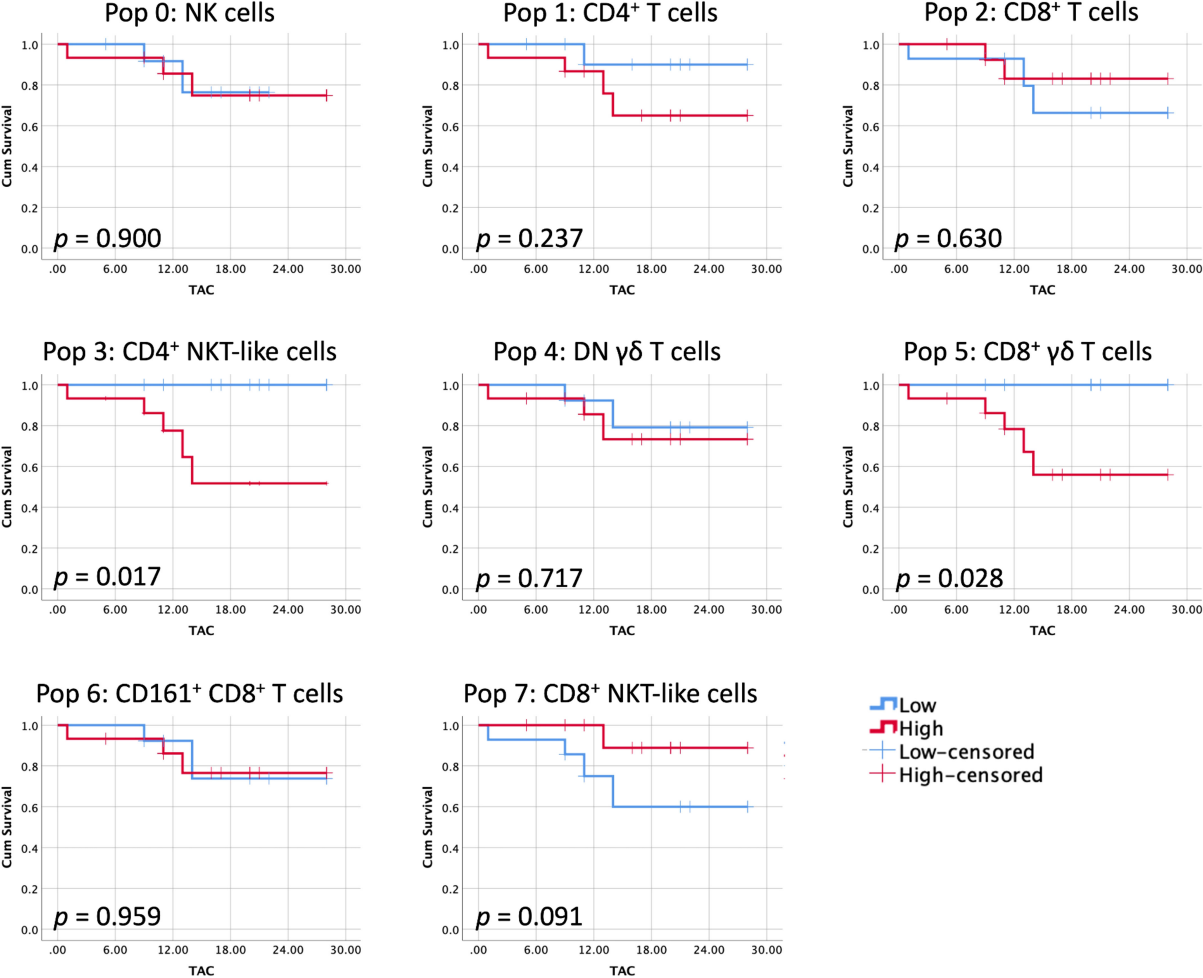
Immune clusters individually associated with patient survival. Kaplan–Meier curves were generated for STS patients stratified into high and low groups based on the median frequency of each identified immune cell population. Censored events are indicated by crosses on the corresponding curves. Log-rank test was used to compare high and low curves, with significance set at *p* < 0.05. *Legend: TAC – time after collection*.

These immune profile classifications capture group-level patterns of population distribution. Survival outcomes were then evaluated at the individual patient level by analyzing subtype frequencies per sample, with subtype-based immune profiles and individual-level survival analyses providing complementary but distinct perspectives on the data. Patients were stratified into ‘high’ and ‘low’ groups based on the median frequency of each identified immune cell cluster, and survival analyses based on TAC were conducted. Kaplan–Meier curves for each population are presented in **Figure 4**. Higher frequencies of CD4^+^ NKT-like cells (*p* = 0.017) and CD8^+^ γδ T cells (*p* = 0.028) were significantly associated with reduced survival. In contrast, a trend toward improved outcomes was observed in patients with higher levels of CD8^+^ NKT-like cells (*p* = 0.091). No significant associations were found for the remaining immune populations.

### 3.4 STS tumors exhibit distinct patterns of CD3^+^ and/or CD56^+^ populations compared to blood

**Figure 5:**
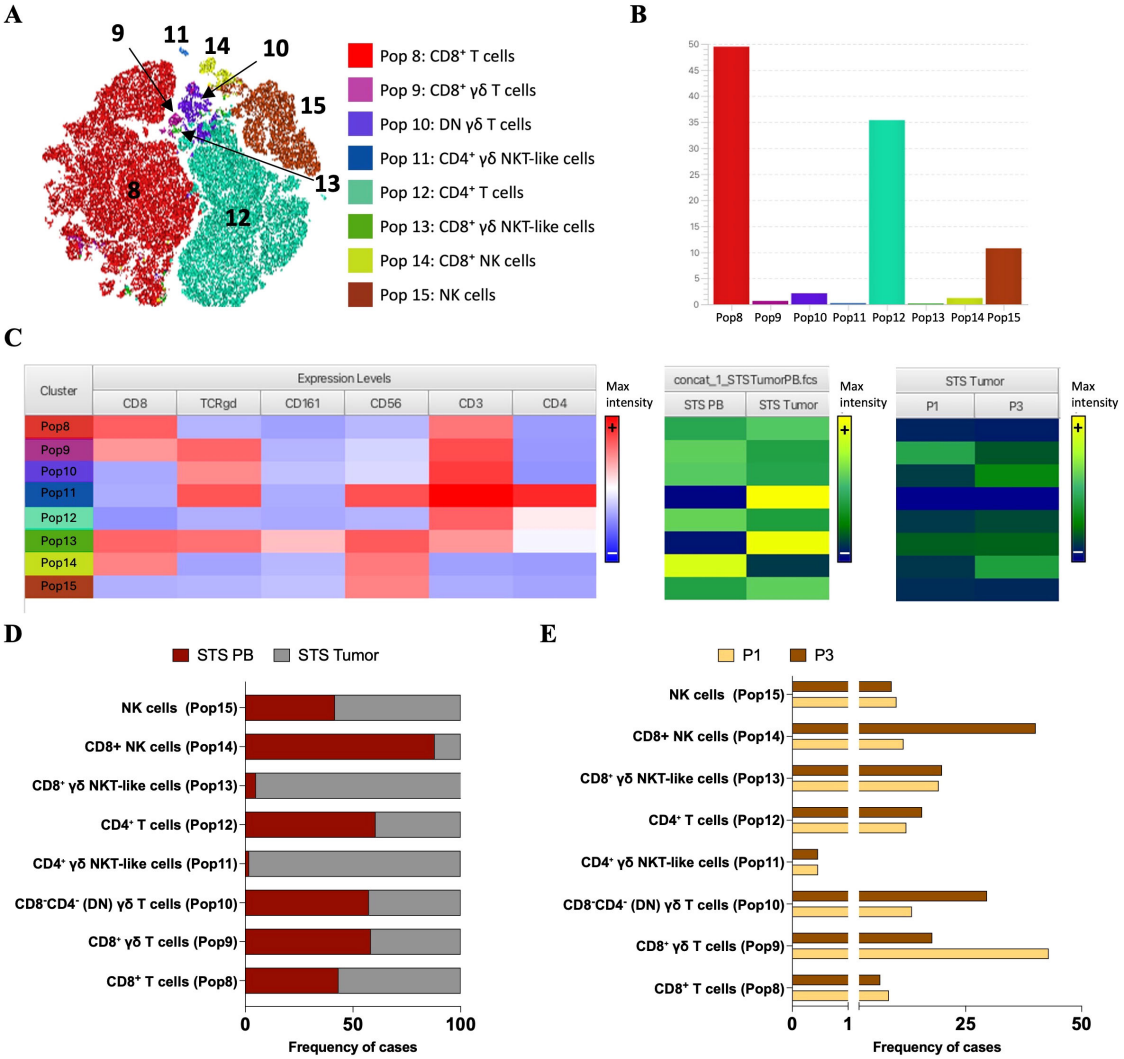
Cluster Explorer analysis reveals differential distribution of immune cell populations between PB and tumor samples from STS patients. Cluster Explorer analysis was performed using FlowSOM and t-SNE results based on the expression of CD3, CD4, CD8, CD56, CD161, and TCR γ/δ, resulting in eight metaclusters representing major immune cell populations. **(A)** t-SNE map displaying FlowSOM-defined clusters based on multiparametric marker expression. Each dot represents a single cell, with colors corresponding to FlowSOM-assigned clusters. **(B)** Bar plot showing the relative frequency of each identified immune population across all samples. **(C)** Cluster Explorer heatmap summarizing marker expression profiles per cluster. Rows represent individual clusters; columns represent surface markers. Color intensities represent median expression levels; (+) indicates higher intensity, whereas (–) indicates lower intensity. **(D)** Bar plot showing the distribution of PB and tumor samples across each immune cluster. **(E)** Bar plot showing the distribution of STS immune subtypes (P1 and P3) per cluster. *Legend: PB – peripheral blood; STS – soft tissue sarcoma; P1 – “immune high”; P3 – “immune low”; Pop 0 – CD8+ T cells; Pop 1 – CD8^+^ γδ T cells; Pop 2 – CD8-CD4- (DN) γδ T cells; Pop 3 – CD4^+^ γδ NKT-like cells; Pop 4 – CD4^+^ T cells; Pop 5 – CD8^+^ γδ NKT-like cells; Pop 6 – CD8+ NK cells; Pop 7 – NK cells*.

Using the same approach applied to PB samples from STS and Ctrl groups, we conducted a new analysis that included the same STS PB samples and a set of 9 STS tumor samples, aiming to explore differences between the peripheral and tumor compartments. It is important to note that not all tumor samples were paired with corresponding PB samples, preventing a true paired analysis. As a result, only four tumor samples could be classified based on the patients’ peripheral immune profiles, specifically, two samples were representative of the P1 (“immune high”) profile and two of the P3 (“immune low”) profile. Following quality control assessment, sample annotation, and manual gating of CD3^+^ and/or CD56^+^ lymphocytes, a single file containing the cells of interest was generated. FlowSOM clustering was then performed on these CD3^+^ and/or CD56^+^ lymphocytes, configured to produce eight metaclusters based on the expression of CD3, CD4, CD8, CD56, CD161, and TCR γ/δ. Cluster Explorer was then employed for dimensionality reduction and annotation. The t-SNE map displays the distribution of cells across the eight clusters identified in PB and tumor samples (**Figure 5A**). The corresponding bar plot summarizes the proportion of events assigned to each cluster, which by default in ClusterExplorer are labeled Pop 0 to Pop 7 and cannot be changed. To avoid confusion with the populations previously defined in blood, these clusters were manually labeled Pop 8 to Pop 15 (**Figure 5B**). In this analysis, Pop 8 and Pop 12 represented the most abundant populations, followed by Pop 15. Pop 9, 10, 11, 13 and 14 accounted for only a minor fraction of the total events.

To characterize the immune phenotypes represented by each cluster, the expression patterns of key surface markers using the Cluster Explorer heatmap were analyzed (**Figure 5C**). Each cluster exhibited a distinct marker expression profile, enabling the inference of putative immune cell identities (**Figures 5A–C**). Pop 8 exhibited high expression of CD3 and CD8, consistent with CD8^+^ T cells. Pop 9 expressed CD3, CD8, and TCR γδ, consistent with CD8^+^ γδ T cells. Pop 10 co-expressed CD3 and TCR γδ, lacking the expression of CD4 and CD8 suggesting DN γδ T cells. Pop 11 expressed CD3, CD4, CD56 and TCR γδ, consistent with CD4^+^ γδ NKT-like cells. Pop 12 co-expressed CD3 and CD4, consistent with CD4^+^ T cells. Pop 13 highly express CD56, CD3, CD8, and TCR γδ, suggesting CD8^+^ γδ NKT-like cells. Pop 14 express CD56 and CD8 suggestive of CD8^+^ NK cells and Pop 15 only express CD56 consistent with NK cells.

Then, we examined the contribution of each sample group to the FlowSOM-defined clusters (Pop 8–Pop 15). Stratification of tumor and PB samples revealed distinct population distributions (**Figures 5C, D**). Tumor-derived events contributed slightly more to CD8^+^ T cells (Pop 8) and NK cells (Pop 15). In contrast, CD8^+^ NK cells (Pop 14) were mainly derived from PB samples, which also contributed modestly to CD8^+^ γδ T cells (Pop 9), DN γδ T cells (Pop 10), and CD4^+^ T cells (Pop 12). Interestingly, although rare, two populations, CD4^+^ γδ NKT-like cells (Pop 11) and CD8^+^ γδ NKT-like cells (Pop 13), were almost exclusively tumor-derived, underscoring their selective presence in the TME and near absence in peripheral blood. Given that only two tumor samples were assigned to the P1 and P3 immune profiles, analyses based on STS profiles are purely illustrative (**Figures 5C, E**).

### 3.5 STS tumors harbor rare CD8+ γδ NKT-like cells absent in peripheral blood

**Figure 6:**
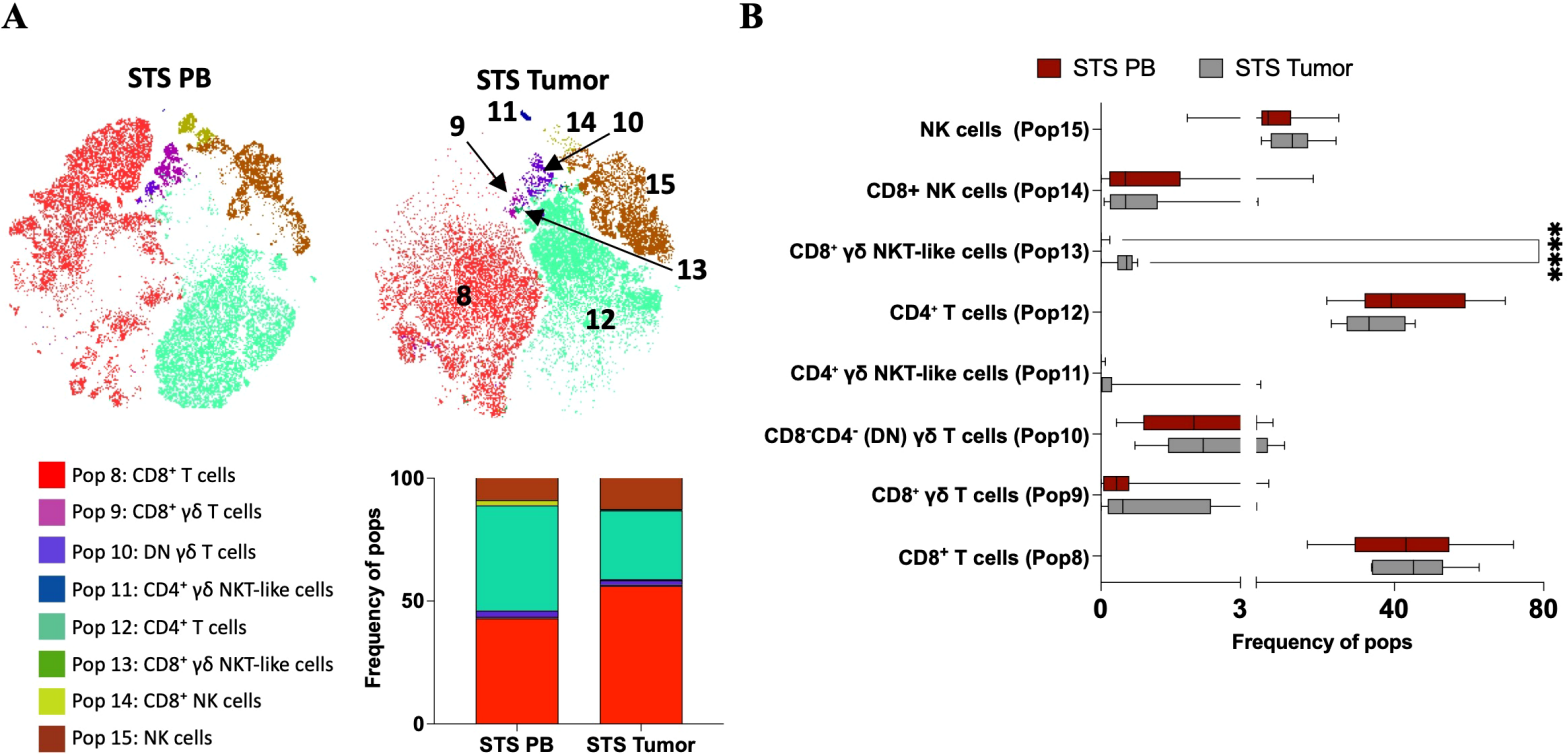
Comparative analysis of immune cluster frequencies in PB and tumor samples from STS patients reveals compartmentalized immune profiles. **(A)** t-SNE plots colored by FlowSOM-assigned clusters, representing PB and tumor samples. Bar plot representation of FlowSOM-defined immune cluster frequencies in all samples and across analyzed groups, PB and tumor samples from STS patients. **(B)** Comparative analysis of cluster frequencies between STS and Ctrl samples. Statistical analysis was performed using the Mann–Whitney U test with Holm-Šídák method to correct for multiple comparisons. Adjusted p-values are reported, with significance set at p < 0.05. Legend: PB – peripheral blood; STS – soft tissue sarcoma; ****p < 0.0001.

The back-gating of the FlowSOM populations for sample-level quantification within PB and tumor samples from STS patients was then performed. **Figure 6A** shows a t-SNE map illustrating the distribution of the eight identified populations in PB and tumor samples, alongside a bar graph displaying their relative frequency contributions within each group. Despite their low frequency, individual sample analysis revealed a consistent and significant presence of CD8^+^ γδ NKT-like cells (Pop 13) in tumor samples (Pop 13 = 0.55, 0.00–0.79) compared with near absence in PB samples (Pop 13 = 0.00, 0.00–0.19, adj p < 0.0001) (**Figure 6B**). Similarly, although not statistically significant, CD4^+^ γδ NKT-like cells (Pop 11) were primarily observed in tumor samples (Pop 11 = 0.02, 0.00–4.22) and were nearly absent in PB samples (Pop 11 = 0.00, 0.00–0.10, adj p = 0.0718). No other statistically significant differences were detected.

Considering both cluster- and individual sample-based analyses, similar distribution patterns were observed, with slightly higher levels of NK cells (Pop 15) and CD8^+^ T cells (Pop 8), and lower levels of CD4^+^ T cells (Pop 12) in tumor samples. For CD8^+^ NK cells (Pop 14), individual sample analysis did not clearly show higher prevalence in PB samples, as median values were similar between PB and tumor; however, variability was higher in PB, with some patients displaying elevated levels. Since event numbers were equalized per group, these high-level samples contributed to the higher prevalence observed in cluster-based analysis. In contrast, CD8^+^ γδ T cells (Pop 9) appeared elevated in more tumor samples at the individual level, whereas cluster-based analysis suggested slight enrichment in PB. Overall, these observations should be interpreted as preliminary, given the limited number of tumor samples.

## 4 Discussion

Our group previously performed a comprehensive analysis of the immune cellular landscape in STS, including myeloid (monocytes, dendritic cells, granulocytes) and lymphoid populations (B cells, NK cells, T cells, as well as Th and Treg subsets), together with immune-related genes and soluble factors (13). That study revealed overall immunosuppression and reduced cytotoxic potential in STS. Moreover, three distinct immune profiles, “immune high” (P1), “immune intermediate” (P2), and “immune low” (P3), were identified and correlated with survival. Patients with higher cytotoxic and lower suppressive factors (“immune high”) showed better outcomes, whereas the opposite pattern (“immune low”) was linked to worse prognosis.

Building on these findings, we hypothesized that impaired anti-tumor activity in STS may also involve cytotoxic lymphocyte populations not captured by conventional approaches, such as unconventional T cells. To test this, we analyzed CD3^+^ and/or CD56^+^ lymphocytes in a subset of patients from the previous cohort using multiparametric flow cytometry, and applied AI-assisted unsupervised clustering to comprehensively map the systemic cytotoxic immune landscape in STS. This approach enabled the identification of less frequent lymphocyte subsets with recognized cytotoxic potential. In parallel, we performed a pilot analysis of tumor samples using the same strategy to obtain preliminary insights into the compartmentalization of these populations.

Consistent with our previous findings, STS patients showed a significant increase in circulating CD8^+^ T cells and a slight decrease in CD4^+^ T cells compared with healthy donors, in line with reports of elevated antigen-specific CD8^+^ T cells in cancer patients (13, 22, 23), which is more evident in P3 patients. No significant association between total CD8^+^ or CD4^+^ T cells and STS immunotypes was observed. However, t-SNE visualization suggested distinct distribution patterns of these cells across P1, P2, and P3 patients, indicating potential differences in CD8^+^ and CD4^+^ T subpopulations among immunotypes. Although these subsets could not be fully characterized here, our earlier study in a larger cohort (13) showed that P3 (“immune low”) patients had higher levels of Th2 cells and lower Th1 cells, whereas P1 patients displayed the opposite profile.

Circulating NK cells were significantly reduced in STS patients, consistent with our previous study (13) and with other reports in cancer, where NK cell depletion, critical for controlling tumor dissemination through elimination of circulating tumor cells (24), has been linked to poorer survival and higher metastatic potential in both hematologic and solid tumors (25–29). Interestingly, although not statistically significant, P3 patients showed a higher frequency of NK cells compared with other immunotypes. Given that P3 is associated with worse prognosis, this finding appears contradictory to the expected protective role of NK cells and instead suggests that, despite their higher frequency, NK cell function may be compromised in these patients. Supporting this, our previous study showed reduced expression of cytotoxic effector molecules such as GZMB and PRF1 in P3 patients, indicating impaired NK cell cytotoxicity.

Among the less represented clusters, STS patients showed a significantly lower frequency of CD161^+^ CD8^+^ T cells. CD161 expression on T cells is associated with innate-like properties relevant to mucosal and tissue immunity (30, 31), and in cancer, their presence in circulation or within tumors has been linked to preserved immune competence (32, 33). Thus, their reduced levels in STS patients are consistent with an impaired immune status. Concatenated analyses indicated that P3 patients contributed disproportionately to this population. Although the median frequency of CD161^+^ CD8^+^ T cells was lower in P3 than in P1, greater variability was observed, with some P3 patients displaying relatively high levels. This discrepancy likely reflects methodological limitations, including unequal group sizes and event equalization in clustering. Nevertheless, taken cautiously, these findings may suggest a compensatory or dysregulated immune response in P3 patients. Alternatively, it may point to altered tissue distribution or functional impairment of these cells in more immunosuppressed individuals.

γδ T cells are innate-like lymphocytes with well-recognized anti-tumor activity (34–36). In this study, we identified two circulating γδ T cell populations: double-negative (DN) γδ T cells and CD8^+^ γδ T cells. DN γδ T cells, which constitute the majority of circulating γδ T cells, are typically associated with immunoregulatory functions (37), whereas the less frequent CD8^+^ γδ T cells are known for strong cytotoxic and pro-inflammatory activity in cancer (38–40). Both populations were similarly represented in STS patients and healthy controls. Within STS, higher levels were observed in P2 patients, although this difference was not statistically significant. Notably, survival analysis revealed that patients with higher frequencies of circulating CD8^+^ γδ T cells had significantly reduced survival. This counterintuitive finding may reflect compensatory immune activation in response to more aggressive disease or an exhausted phenotype limiting effective anti-tumor function.

Two clusters of NKT-like cells were identified: CD4^+^ and CD8^+^ NKT-like cells. These T lymphocytes express CD56 and other NK cell markers, with frequencies increasing with age (41, 42). They combine conventional T cell functions with NK-like cytotoxicity via receptors such as NKG2D, enabling rapid cytokine production and potent cytotoxic responses. Altered frequencies or phenotypes of NKT-like cells have been linked to immune dysregulation in several malignancies (43–48), and CD56 expression is associated with enhanced anti-tumor activity (49). CD8^+^ NKT-like cells are generally considered cytotoxic, whereas CD4^+^ NKT-like cells may exhibit helper or regulatory functions (50). In this study, CD8^+^ NKT-like cells tended to be more frequent in healthy controls than in STS patients, though not significantly at the individual level. Within STS, they were enriched in the P1 (“immune high”) subgroup and showed a trend toward higher frequencies in patients with better survival. In contrast, CD4^+^ NKT-like cells displayed no major differences across groups or immune profiles but were associated with poorer survival when present at higher frequencies. These findings underscore the heterogeneity of NKT-like cells in shaping systemic immune competence and disease outcomes in STS. It is conceivable that CD8^+^ NKT-like cells could be therapeutically expanded using cytokine-induced approaches, similar to cytokine-induced killer (CIK) cell strategies (45, 51), although their functional potential in STS remains untested.

Additionally, a pilot analysis was performed on a small subset of STS tumor samples using the same strategy to gain preliminary insights into tumor-infiltrating lymphocytes (TIL, CD3^+^ and/or CD56^+^ cells). Comparing with PB, tumor samples showed slightly higher levels of CD8^+^ T cells and NK cells, and lower levels of CD4^+^ T cells, consistent with the established role of CD8^+^ T cells and NK cells as key effectors in tumor infiltration and cytotoxic activity (52–54).

When analyzing peripheral blood from STS and control patients, only a single NK cell population was identified. CD8 expression within this population was moderate, insufficient to define a distinct CD8^+^ NK cell cluster. In contrast, analysis of tumor-infiltrating lymphocytes (TIL) revealed two NK cell populations based on CD8 expression. Although little is known about CD8^+^ NK cells, evidence suggests that transient CD8 expression marks a highly functional state, while sustained expression may indicate reduced activity and an inhibitory role in NK function (55, 56). In cluster-based analysis, PB-derived events contributed predominantly to the CD8^+^ NK cell population. However, individual sample analysis showed similar median frequencies in both PB and tumor samples, with considerable variability among PB samples; some patients exhibited higher frequencies, which disproportionately influenced the cluster analysis due to unequal group sizes. Illustrative examples from P1 and P3 patients suggested higher infiltration of CD8^+^ NK cells in P3 tumors, though no conclusions can be drawn. These observations highlight the need to further investigate the role of CD8^+^ NK cells in STS tumors.

DN γδ T cells and CD8^+^ γδ T cells were also identified in tumor samples. In cluster analysis, PB samples contributed slightly more to these populations; however, individual sample analysis revealed substantial variability in CD8^+^ γδ T cell frequencies across tumors. Notably, illustrative examples from P1 and P3 patients showed higher infiltration of CD8^+^ γδ T cells in the P3 tumors. Although these findings must be interpreted with caution, they are consistent with peripheral blood data, where elevated CD8^+^ γδ T cell levels correlated with reduced survival. This raises the possibility that tumor-infiltrating CD8^+^ γδ T cells may also hold prognostic significance. A larger cohort analysis will be necessary to validate these preliminary observations and to clarify whether CD8^+^ γδ T cells represent a compensatory, dysfunctional, or prognostically relevant population in STS.

Interestingly, despite their low frequency at the individual level, two γδ NKT-like subpopulations, CD8^+^ and CD4^+^ γδ NKT-like cells, were identified in tumors but were nearly absent in circulation. CD8^+^ γδ NKT-like cells, in particular, were consistently observed across the tumor samples analyzed, even though the sample number was limited. Although little is known about these cells, previous studies suggest that γδ NKT-like cells can exert potent cytotoxic effects against solid tumors such as squamous cell carcinoma and produce high levels of IFN-γ (40, 57–59). Increased frequencies have also been reported in malignant compared with normal liver tissue (60, 61). Their selective enrichment in STS tumors could therefore reflect local recruitment or expansion within the tumor microenvironment, potentially contributing to antitumor immune responses. Nevertheless, the very small number of tumor samples analyzed restricts the robustness of this observation. These findings should thus be regarded as preliminary and interpreted with caution. Even so, they highlight potentially important tumor-associated populations that warrant further investigation in larger STS cohorts.

This study has several limitations that should be acknowledged. First, concatenated analyses, performed without an equal number of samples per group, normalize event numbers at the group or subtype level but do not guarantee equal contributions from all patients. This can lead to discrepancies when comparing group-level and per-sample analyses, highlighting the need to consider both approaches to properly capture inter-patient heterogeneity. Second, the overall sample size was small, especially for tumor samples, which restricts the generalizability of the findings. Third, peripheral blood samples were collected at different time points relative to diagnosis and disease stage, introducing variability that may affect immune measurements and complicate comparisons across patients. In addition, not all tumor samples could be matched to a peripheral immune profile, and some were not collected in parallel with blood, limiting paired analyses and reducing the ability to directly link systemic and intratumoral features. Finally, the lack of functional characterization of key immune subsets, particularly those associated with survival, remains a major gap. Future studies with larger, longitudinal cohorts and standardized sampling, coupled with functional assays of cytokine production, cytotoxic activity, inhibitory receptor expression, and soluble mediators, will be essential to validate these findings and clarify the prognostic and therapeutic relevance of the immune populations identified.

**Figure 7:**
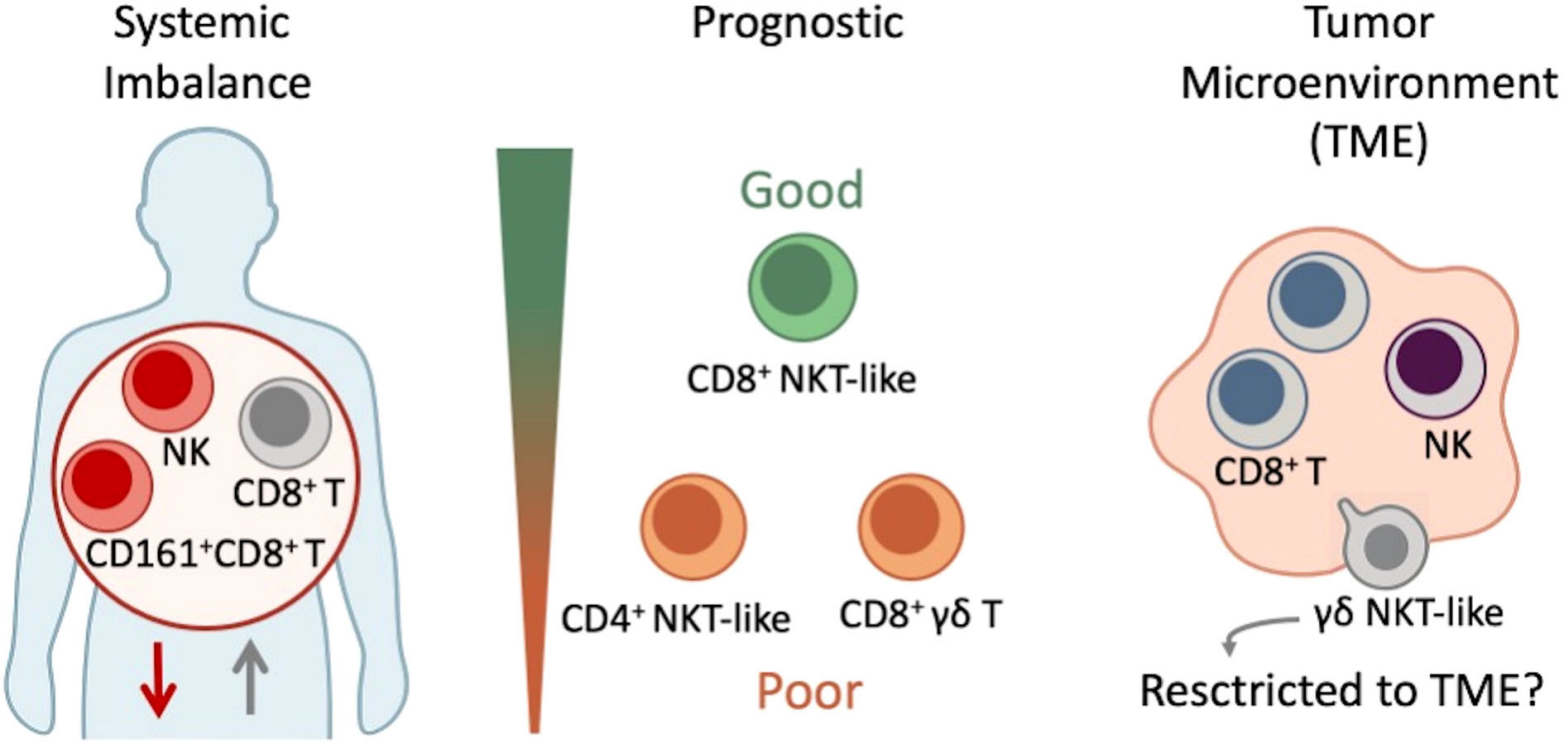
Schematic representation of immune alterations in soft tissue sarcoma (STS). Patients displayed systemic immune imbalance with increased CD8^+^ T cells and reduced NK cells and CD161^+^ CD8^+^ T cells, consistent with overall immunosuppression. Prognostic associations were observed for unconventional populations: elevated CD8^+^ γδ T cells and CD4^+^ NKT-like cells correlated with poorer survival, whereas CD8^+^ NKT-like cells were enriched in immune-competent patients and associated with better outcomes. Pilot tumor analyses revealed γδ NKT-like cells that were nearly absent in circulation but present within the tumor microenvironment, suggesting potential selective enrichment.

This study expands the understanding of the cytotoxic immune landscape in STS by integrating multiparametric flow cytometry with unsupervised clustering to identify both conventional and unconventional lymphocyte populations, with the main findings summarized in **Figure 7**. Consistent with previous findings, STS patients displayed overall systemic immunosuppression, with reduced NK cells and CD161^+^ CD8^+^ T cells, and distinct distribution patterns of CD4^+^ and CD8^+^ T cells across immune subgroups. Notably, CD8^+^ γδ T cells emerged as a paradoxical population, enriched in some patients and associated with poorer survival, suggesting compensatory activation or dysfunction in advanced disease. In contrast, CD8^+^ NKT-like cells showed a trend toward favorable outcomes, while CD4^+^ NKT-like cells were linked to poorer prognosis, underscoring the functional heterogeneity of these populations. Pilot tumor analyses further revealed differences between blood and tumor compartments, including the detection of CD8^+^ NK cells, variable infiltration of CD8^+^ γδ T cells, and the selective enrichment of γδ NKT-like subsets within tumors. Although preliminary and limited by small sample size, these findings highlight tumor-associated populations that may contribute to disease progression or control. Together, these results reinforce the role of cytotoxic and unconventional lymphocyte subsets in shaping systemic and intratumoral immunity in STS. Larger, longitudinal studies integrating functional assays will be essential to validate their prognostic relevance, clarify their contribution to tumor control, and assess their potential as therapeutic targets.

## Data Availability

The raw data supporting the conclusions of this article will be made available by the authors, without undue reservation.
