# Supplementary material for: AI-assisted peripheral immune profiling reveals unconventional lymphocyte signatures associated with prognosis in soft tissue sarcoma patients
Source: Front Immunol. 2025 Oct 27;16:1677408. doi: 10.3389/fimmu.2025.1677408 (PMC12597948; doi:10.3389/fimmu.2025.1677408)

Supplementary Material

Supplementary Table S1. Demographic and clinical data of STS patients and controls samples included in the study.

Characteristic, unit	N (%) or mean (SD)	
	Ctrl	STS
Samples	25	29 (100%)
Age, years	49 ± 10	54 ± 14
Sex (% of females)	15 (60%)	16 (55%)
Disease status		
Non-metastatic		14 (48.2%)
Primary		13 (45.8%)
Recurrence		1 (3.4%)
Metastatic		15 (51.7%)
Primary		13 (45.8%)
Recurrence		2 (6.9%)
Primary anatomical localization		
Extremity (Lower limb)		14 (48.2%)
Trunk (not retroperitoneal)		8 (36.4%)
Thorax		4 (13.8%)
Pelvis		2 (6.9%)
Liver		1 (3.4%)
Trunk unspecified		1 (3.4%)
Retroperitoneal		3 (10.3%)
Gynecological region		4 (13.8%)
Uterus		3 (10.3%)
Spermatic cord		1 (3.4%)
Lineage of cell differentiation		
Leiomyosarcoma		6 (20.7%)
Liposarcoma		4 (13.8%)
Undifferentiated sarcoma		7 (24.1%)
Synovial sarcoma		6 (20.7%)
Other		6 (20.7%)
Malignant peripheral nerve sheath tumor		2 (6.9%)
Hemangiosarcoma		1 (3.4%)
Clear cell sarcoma		1 (3.4%)
Embryonal sarcoma		1 (3.4%)
Endometrial stromal sarcoma		1 (3.4%)
Therapy		
Anthracycline-based therapy		7 (16.4%)
Anthracycline-based therapy followed by trabectedin-based therapy		9 (34.5%)
Trabectedin-based therapy		3 (14.5%)
Other		3 (20%)
Not applicable		7 (14.5%)
Peripheral Immune Profile		
P1 – “Immune high”		9 (56.4%)
P2 – “Immune intermediate”		8 (36.4%)
P3 – “Immune low”		12 (7.3%)

Supplementary Table S2. Detailed description of the fluorochrome-conjugated monoclonal antibodies used to perform multiparametric flow cytometry analysis.

Antibody	Conjugate	Clone	Brand	Cat#	RRDI
CD3	V450	ICHT1	BD Horizon™	561416	AB_10612021
CD4	V500	RPA-T4	BD Horizon™	560768	AB_1937323
CD8	APC-H7	SK1	BD™	641400	AB_1645736
CD45	FITC	HI30	BD Pharmingen™	555482	AB_395874
CD56	PerCP-Cy5.5	B159	BD Pharmingen™	560842	AB_2033964
CD161	PE-Cy7	HP-3G10	Biolegend™	339918	AB_11126745
TCR γ/δ	PE	B1	Biolegend™	331210	AB_1089218

Supplementary File S3. Representative images of the manual gating strategy used to identify lymphocyte populations expressing CD3 and/or CD56 in peripheral blood **(A)** and tumor **(B)** samples using FlowJo® v.10.9 software. First, FlowAI plugin was used to remove low-quality events. **A)** lymphocytes were gated based on FSC-A vs. SSC-A, doublets were excluded, and $CD3^{-}CD56^{-}$ events were used with a "make not-gate" strategy to isolate $CD3^{+}$ and/or $CD56^{+}$ populations. **B)** $CD45^{+}$ events were selected, doublets excluded, lymphocytes gated by FSC-A vs. SSC-A, and the same "make not-gate" approach was applied to $CD3^{-}CD56^{-}$ events to identify $CD3^{+}$ and/or $CD56^{+}$ cells.

A

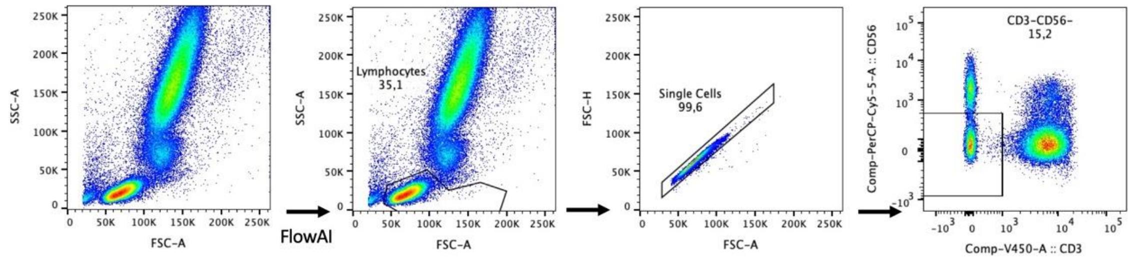

B

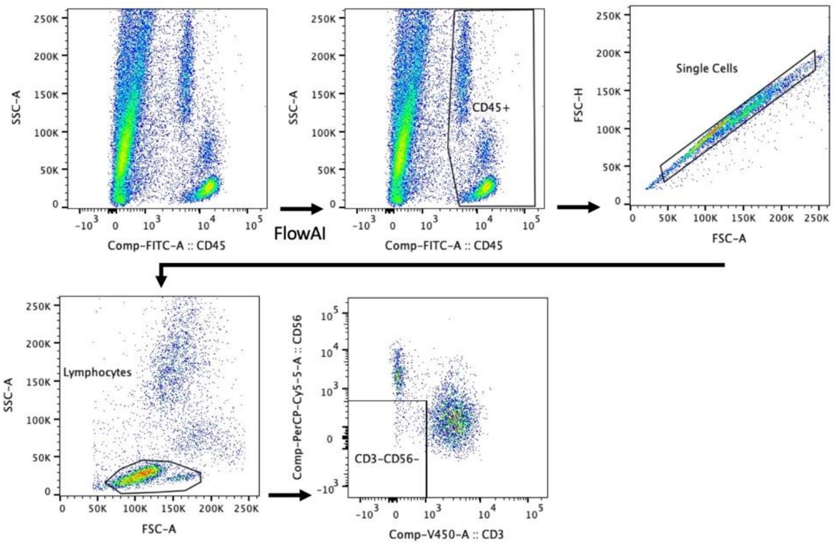

Supplementary File S4. Representative images demonstrating the selection of experimental groups in the concatenated files for peripheral blood **(A, B)** and tumor **(C, D)** samples using FlowJo® v10.9 software.

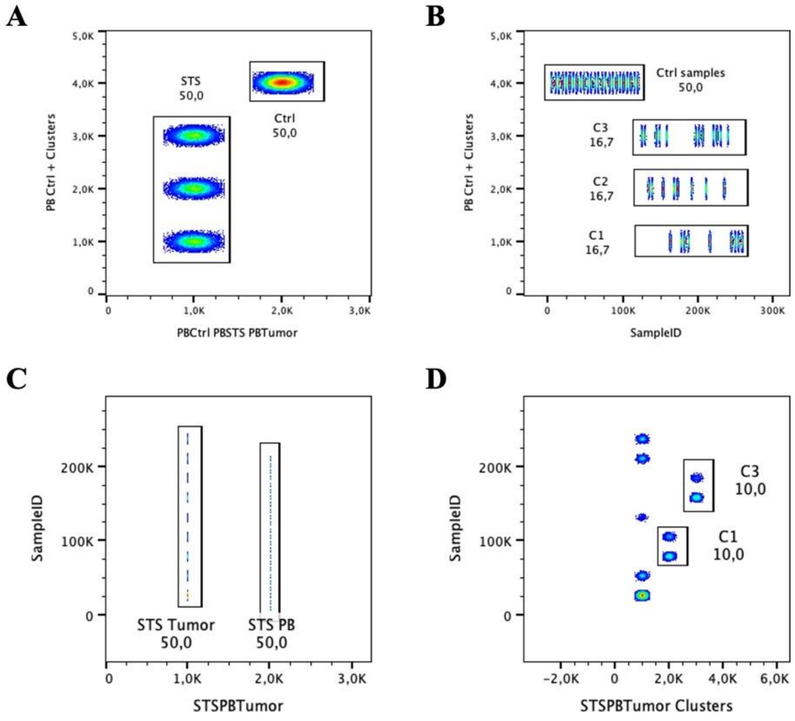

Supplementary File S5. Survival analysis of STS peripheral immunotypes. Kaplan–Meier curves were generated for STS patients categorized according to previous stablished peripheral immune profiles, P1 (“immune high”), P2 (“immune intermediate”), and P3 (“immune low”) immunotypes. Survival analysis was assessed using time-to-event from the date of sample collection to either the date of death or the end of follow-up, referred to as TAC (months). Censored events were identified as a cross in the respective curves. Log-rank test was used to compare high and low curves, with significance set at $p < 0.05$. *Legend: TAC – time after collection.*

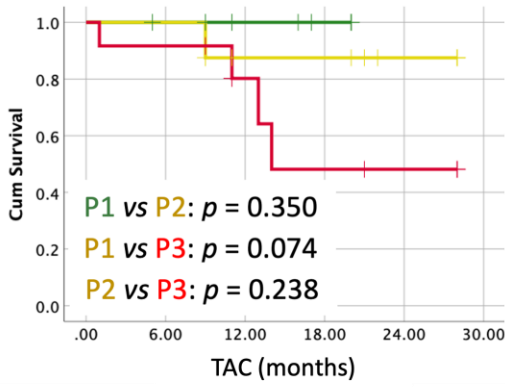

Supplement: Supplementary file 1 [file DataSheet1.pdf]
